# Supplementary material for: Sustained elevation of soluble B- and T- lymphocyte attenuator predicts long-term mortality in patients with bacteremia and sepsis
Source: PLoS One. 2022 Mar 21;17(3):e0265818. doi: 10.1371/journal.pone.0265818 (PMC8936450; doi:10.1371/journal.pone.0265818)
Supplement: S1 Table — Baseline and clinical characteristics in patients with ≤1 and ≥2 missing samples. (PDF) [file pone.0265818.s001.pdf]

S1 Table. Missing sample analysis

	≤1 missing sample (N =48)	≥2 missing samples (n=60)
Age median (IQR)	69 (57-78)	73 (64-84)
sex male	29 (60)	30 (50)
SOFA score median (IQR)	1 (1-3)	2 (1-3)
Charlson score median (IQR)	1 (0-2.75)	1 (0-2)
Mortality 28 days	1 (2)	4 (7)
Mortality 90 days	5 (10)	6 (10)
Mortality 1 year	10 (21)	14 (23)
E. coli	9 (19)	16 (27)
S. aureus	13 (27)	14 (23)
S. pneumoniae	14 (29)	15 (25)

Data is presented as N (%) unless otherwise stated.

p-value
0.03
0.28
0.91
0.37
0.38
0.94
0.76
0.33
0.65
0.63
