# Supplementary material for: Sustained elevation of soluble B- and T- lymphocyte attenuator predicts long-term mortality in patients with bacteremia and sepsis
Source: PLoS One. 2022 Mar 21;17(3):e0265818. doi: 10.1371/journal.pone.0265818 (PMC8936450; doi:10.1371/journal.pone.0265818)
Supplement: S2 Table — (PDF) [file pone.0265818.s002.pdf]

		90-day mortality				1 year mortality			
	**BSI n=96**	**Events[1]**	**Rate[2]**	**Adjusted[3]**		**Events**	**Rate[2]**	**Adjusted[3]**	
**Day 1-2**				**HR (95% CI)**	**p**			**HR (95% CI)**	**p**
log10 sBTLA, per unit	96	12		112 (3.8-3297)	<0.01	15		11 (0.97-126)	0.05
sBTLA<11	40	3	32	Ref		5	14	Ref	
sBTLA≥11	56	9	70	7.0 1.3-38)	0.03	15	33	5.9 (1.6-22)	<0.01
	**BSI n=85**	**Events[1]**	**Rate[2]**	**Adjusted[3]**		**Events**	**Rate[2]**	**Adjusted[3]**	
**Day 7**				**HR (95% CI)**	**p**			**HR (95% CI)**	**p**
log10 sBTLA, per unit	85	10		23 (1.1-482)	0.045	17		30 (3.0-312)	<0.01
sBTLA<11	42	1	11	Ref		2	5	Ref	
sBTLA≥11	43	9	102	18 (1.9-175)	0.01	15	47	15 (2.9-81)	<0.01

[1] Number of deaths

[2] Mortality rate defined as deaths per 100 person years at risk

[3] For age, sex, delta-SOFA, comorbidity (Charlson score (0, 1-2, ≥3), and immunosuppression at baseline
