# Supplementary figures and images for: Sustained elevation of soluble B- and T- lymphocyte attenuator predicts long-term mortality in patients with bacteremia and sepsis
Source: PLoS One. 2022 Mar 21;17(3):e0265818. doi: 10.1371/journal.pone.0265818 (PMC8936450; doi:10.1371/journal.pone.0265818)

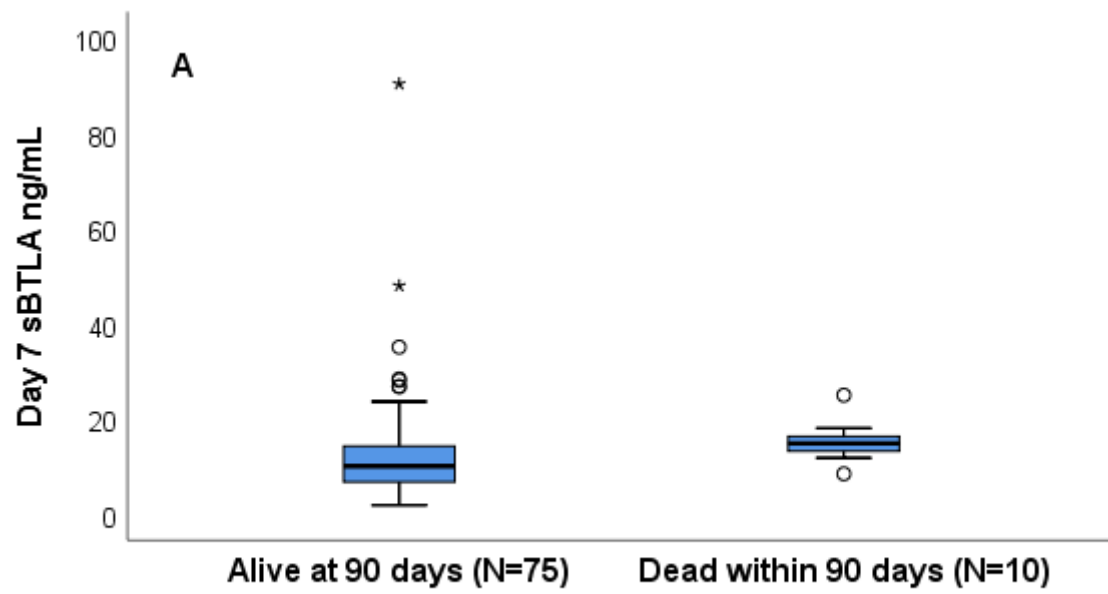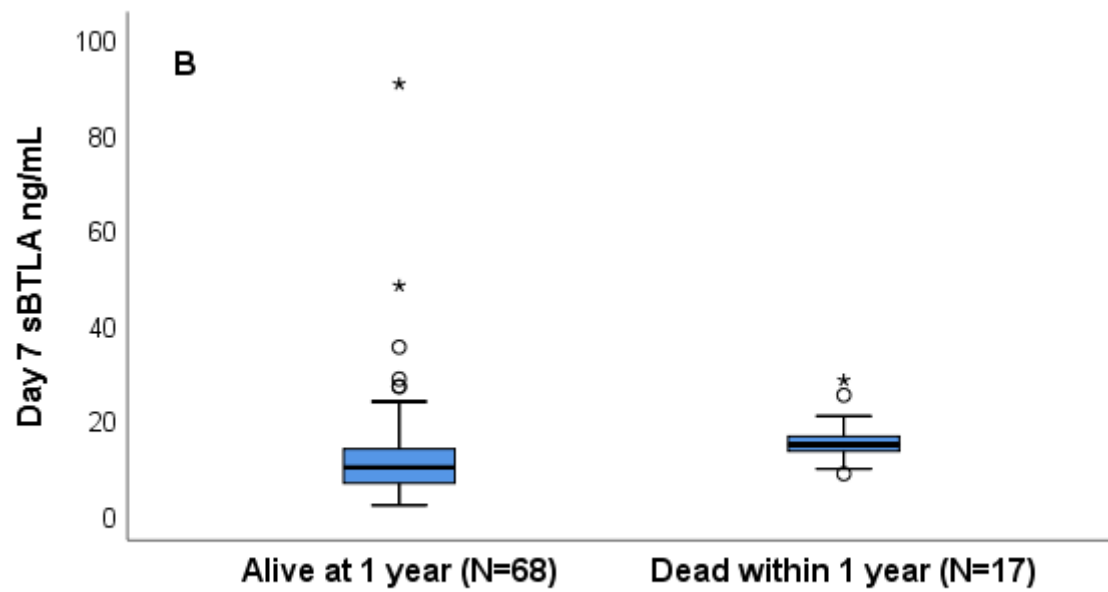

Supplement: S1 Fig — (A) sBTLA on day 7 in patients alive at, or dead within 90 days post hospital admission (B) sBTLA on day 7 in patients alive at or dead within 1 year post hospital admission. (PDF) [file pone.0265818.s003.pdf]
