# Supplementary material for: Sustained elevation of soluble B- and T- lymphocyte attenuator predicts long-term mortality in patients with bacteremia and sepsis
Source: PLoS One. 2022 Mar 21;17(3):e0265818. doi: 10.1371/journal.pone.0265818 (PMC8936450; doi:10.1371/journal.pone.0265818)
Supplement: Attachment — Submitted filename: Response to reviewers.docx [file pone.0265818.s005.docx]

*We would like to thank the Editor, Dr Adrish, and the reviewers for reviewing our manuscript, and for Your interesting and relevant questions, and invaluable suggestions. We have taken them into account to improve our manuscript. Our responses follow directly below each comment. We refer to pages and lines in the clean revised manuscript.*

**Response to the Editor:**

*We have ensured that the manuscript meets PLOS ONE´s style requirements. As requested, we have provided additional information on the participant recruitment method, i e. the recruitment date range, a statement regarding the sample, and a description of how participants were recruited (page 5, lines 13-22 in the revised manuscript, clean version). A full ethics statement has been added to the Methods section (page 5 line 2 to page 6 line 2). The phrase data not shown have been removed from the manuscript. We have supplied all data in the missing data analysis in a supplemented table (S1 Table). On page 11 (Caption "sBTLA in Patients and controls") the sentence stating that there was not an association with sex was removed, as it is not a core part of the results and the mortality analysis are sex-adjusted. On page 15, line 2, we refer to immunosuppression-adjusted mortality analyses in S2 Table, which has been added as supporting information, and finally, on page 16, data previously stated as not shown have been added to Fig 6 legend. Captions for supporting information files have been added to the end of the manuscript.*

Reviewer #1: This manuscript technically sound, and the data support the conclusions. The sample size seems acceptable .Data analysis was appropriate also it seems that all data are available. Various groups of patients with different types of infection was included in study , but the main limitation is the number of patients and it seems that we need more patients and also an study with a larger sample size

**Response to reviewer #1.**

*Thank you for your comments. We acknowledge that the main limitation of this study is the sample size (page 20, line 5), and that our results need to be validated in a larger cohort (page 21 lines 10-12).*

**Reviewer #2: Thank you for the invitation to review this manuscript.**

The authors present data on serial sBTLA levels in a 2011-14 patient cohort admitted with acute illness due to confirmed bloodstream infection. Their principal finding was of an association between persistently elevated sBTLA levels at day 7 and mortality at 90 days and 12 months.

Comments

1. Could the authors please include a concise description of what is known about the biology of sBTLA? Are lymphocytes / specific lymphocyte subsets the origin? What differentiates cell surface bound from soluble BTLA? What is the fate and intravascular half-life of sBTLA What effector cells does sBTLA act upon and to what effect? How is individual age known to affect the production and kinetics of sBTLA?

***Response:*** *First, we would like to thank You for scrutinizing our manuscript, and for Your many important suggestions. We have expanded on the origin and possible biological effects of sBTLA in the introduction and discussion. Soluble BTLA lacks the transmembrane domain of its cell-bound counterpart and is produced either by proteolytic shedding or alternative mRLA splicing (page 4 lines 15-17). Here, a novel review by Kahn et al, covering soluble checkpoint molecules was added for reference (reference 20).*

*sBTLA transcripts has been identified in B cells and T cells (CD4+ CD8+) (page 4, lines 85-86). The target cells, and immunoregulatory significance of soluble BTLA is only partly known, and the results of previous experimental studies are addressed in the Discussion (page 19 lines 10-15).*

*We have found no reference on the kinetics of sBTLA in the circulation, but hopefully, the ongoing study on a BTLA fusion protein TAB004 (NCT04137900) will give information on this.*

*With regards to your question on age, up until the submission of this manuscript, the only reference answering to this was a Chinese study on healthy subjects that reported increasing serum sBTLA with age (Reference 36). However, there is a just recently published study reporting no difference in sBTLA among cancer patients older or younger than 67 years (Reference 31). That study also evaluated the association between sBTLA and PBMC expression of BTLA on a subset of patients, and found that serum levels of s BTLA strongly correlated to the percentage of circulating CD3 + CD8 expressing BTLA (page 20. line 1-4).*

2. Could the authors please account for the apparent slow recruitment of 116 patients over 4 years and the 7 year gap between the close of recruitment and the submission of this paper?

***Response:*** *We acknowledge that study recruitment was slow. Reasons to this was 1) The number of beds at Örebro University Hospital, and the Department of infectious diseases (460 and 28 respectively) during the recruitment period, and 2) Enrolment was only possible during office hours, as study samples were drawn by study nurses who did not work on weekends.*

*Several scientific questions related to bacterial etiology and immune status have been addressed based on study material from this patient cohort. BTLA-analyses were performed in 2019. The ongoing pandemic has delayed manuscript submission.*

3. Only 14 (13%) of patients were admitted to ICU during their acute hospital admission. Where any patients not admitted to ICU due to poor prognosis or for any other reason despite apparent indications for such an admission? What were the reasons for the ICU admission - specifically, how many were the consequence of the development of septic shock or acute severe organ(s) dysfunction due to the BSI that had precipitated hospital admission? How did ICU admission affect short and long term mortality risk?

***Response:*** *The reason for all ICU admissions was need of organ supportive therapy such as vasoactive drugs or mechanical ventilation, due to sepsis (page 9, line 14-16). 3 patients were not admitted to ICU due to limitations of treatment, (page 12 rows 10-12). Please see response to comment #6 for more details on mortality. Fisher´s Exact Test showed that ICU admission did not statistically significantly affect day 28 (p=0.5), day 90 (p=0.2) or 1 year (0.5) mortality.*

4. I am surprised that the distribution of included patient ages is parametric, I would have expected it to be negatively skewed and reported as a median [IQR] - could the authors comment

***Response:*** *Age was indeed not normally distributed in the study cohort, although the median was equal to the mean. We thank you for your comment, and have changed the description of age in Table 1 (page 10).*

5. In Figure 1, the median value of sBTLA does appear to fall progressively over the 28 day period but by a very small increment compared to the range of measured values. If the authors analysed the delta sBTLA, and / or delta sBTLA as a percentage of the peak value, for each individual, does this provide a more insightful picture of the evolution of sBTLA levels? For example, percentage drop from peak value of both CRP and PCT are emerging as viable stopping criteria for antimicrobial therapy - hence the question.

***Response:*** *Thank you for this interesting and relevant question. There is a wide distribution of sBTLA among patients at each time-point, and as you point out, for a biomarker to be applicable in a clinical setting we need more information about dynamics on an individual level.*

*The median peak day of sBTLA was day 1-2, i.e. the day of enrollment, and the median percentage drop in sBTLA on day 28 from peak concentration was 39.7% (IQR 21.7 -56.8). At the other time-points the median percentage drop from peak sBTLA was: Day 3; 11.8% (IQR -9.8 to 22.9), day 7; 23.7% (IQR 5.6 -39.0, day 14; 32.3% (IQR 16.7 -51.4).*

*We did however choose to use Mixed Model analysis, since it accounts for missing data statistically. We have added more information to the presentation of the result of the Mixed Model analysis to provide quantitative information of the decrease in sBTLA over time (page 11 rows 10-13) “The linear mixed model showed that mean sBTLA decreased by 34.1% (95% CI 13.7 to49.6) from admission to day 28 in subjects with BSI, with a mean decrease by 7.6% (95% CI 4.7 to 10.5) per time point”. Note that these figures correlate rather well to the decrease from peak sBTLA as described above.*

6. The mortality data is fascinating, but requires more information. I presume that cause of death / death certification information is a matter of public record in Sweden? As such, could the authors please provide this data and factor it into their discussion. What does this data tell us about the apparent association between prolonged immune dysregulation and the pathology that causes apparent "premature" death? Or, does this data suggest that the index BSI itself occurred because of an occult pathology that would go on to cause death in the succeeding 12 months?

***Response:*** *We did not have ethical permission to run our data through the public record of cause of death. Therefore, we report and refer to all-cause mortality in the manuscript. 7 patients died as a result of uncontrolled infection during hospital care for the index BSI, out if which 3 were not admitted to intensive care due to treatment limitation as described above. The association between persistent elevation of sBTLA and increased mortality does not per se imply a causal link, but raises the question if, and how sBTLA modifies the immune response. Indeed, it is possible that elevated sBTLA reflects a pathology associated with premature death, which was present before the BSI (page 17, lines 5-8 and page 21 lines 4-7).*

7. Given the association / correlation with widely used existing biomarkers, can the authors comment or speculate on the value of measuring sBTLA levels in addition to, or instead of CRP, for example. Furthermore, could the authors comment on the correlation between sBTLA levels and neutrophil : lymphocyte ratio, which is emerging as a more useful marker than absolute values of either?

***Response:*** *We describe the value of adding sBTLA to immune profile assessment in more detail in the Discussion in the revised manuscript (page 17 lines 11-15, and page 21 lines 10-12). We found no association between sBTLA and neutrophil to lymphocyte count ratio (p=0.26). This result has been added to the results section in the revised manuscript, page 15 lines 12-13).*

8. Could the authors provide a figure that shows the differences in peak, day 7, and day 7 as a percentage of peak in sBTLA levels in survivors verses non-survivors at 28 days, 90 days and 12 months?

***Response:*** *Thank you for this suggestion to increase clarity on the mortality data. Since the focus of the mortality analyses was on day 90 and 1 year mortality, we have added a figure on day 7 sBTLA in survivors and non-survivors day 90 and 1 year as a supplementary figure in the revised manuscript (****S1 Figure****).*

9. Could the authors please add more detail to their suggestions regarding what implications their findings should have in terms of the next questions / hypotheses research into sBTLA and other markers of immunosuppression should aim to address?

***Response:*** *Thank you for this suggestion. We have expanded upon this issue at the end of the Discussion (page 21 line 7-12) in the revised manuscript.*

Reviewer #3: Dear Sirs,

I read both your papers with great interest.

Regarding the paper in review process: the new research actually expends your previous results, regarding sBTLA and sepsis/septic shock patients to the BSI patents. Were some of the patients in both studies? If so, please acknowledge that in the new study (same 31 patients in the control, or just a coincidence?)

I have some concerns regarding the fact that the statistics is too complex, and I think it should be validated by a statistician.

Row 93-95- in introduction do you state your previous research, or did you include a conclusion statement in introduction?

You have a lot of missing data, not only at admission time, but also during the data collecting process. How do you explain this fact? Do you have a table with the demographic data of the controls? It would be interesting to compare their data with patients' data.

You have more than 100 patients in the patients' group and only 31 in the controls. Why did you decide do compare uneven groups?

**Response to Reviewer #3:**

*Thank You for Your most valuable comments on our manuscript.*

- *We used the same control group as in a previous study on soluble checkpoint molecules. Samples from controls were collected in parallel with enrolment of BSI study subjects. ELISA analyses on control plasma have been re-run in association with the samples from patients with BSI. As opposed to the previous study, we here analysed control samples not only from day 0, but also day 28. sBTLA in controls did not differ significantly on day 0 compared to day 28 (p=0.75) (related samples analyses).*
- *All statstical calculations have been validated by the co-author AM, who is a biostatistician.*
- *At the end of the introduction we state our aim and the main finding of the present study accordning to PLOS ONE manuscript guidelines:”Conclude with a brief statement of the overall aim of the work and a comment about whether that aim was achieved”. It is possible that this was a misinterpretaion of the instructions on our part. If so, we will delete the last paragraph.*
- *We had ethical approval to sample all patients that were blood cultured on hospital admission, which was in fact before study enrolment. Due to the prolonged period of enrolment this routine failed. Therefore, we did not include day 0 measurements in mortality analyses. There was missing data over time due to patients not showing up to scheduled follow-up visits. We therefore performed missing data analysis as described in the Statistics section, (page 7 lines 12-15), and found that apart from being older, patients with two or more missing samples did not differ significantly in baseline characteristics or short- and long term mortality. For clarification the results of the missing data analysis can be found in a supplementary table (S1 Table).*
- *One aim of the collection of the patient cohort was to address scientific questions related to bacterial etiology, and the size of the control group is comparable to the number of patients in the main bacterial etiology groups (E. coli, S. aureus and S. pneumoniae). We also did not expect sBTLA plasma concentrations to be widely distributed in healthy controls.*
- *In the revised manuscript we have added control demographics to Table 1.*
